# Lack of functional specialization of neurons in the mouse primary visual cortex that have expressed calretinin

**DOI:** 10.3389/fnana.2014.00089

**Published:** 2014-09-04

**Authors:** Daniela Camillo, Christiaan N. Levelt, J. Alexander Heimel

**Affiliations:** ^1^Cortical Structure and Function Group, Netherlands Institute for NeuroscienceAmsterdam, Netherlands; ^2^Molecular Visual Plasticity Group, Netherlands Institute for NeuroscienceAmsterdam, Netherlands

**Keywords:** calretinin, visual cortex, orientation tuning, spatial frequency, interneurons

## Abstract

Calretinin is a calcium-binding protein often used as a marker for a subset of inhibitory interneurons in the mammalian neocortex. We studied the labeled cells in offspring from a cross of a Cre-dependent reporter line with the CR-ires-Cre mice, which express Cre-recombinase in the same pattern as calretinin. We found that in the mature visual cortex, only a minority of the cells that have expressed calretinin and Cre-recombinase during their lifetime is GABAergic and only about 20% are immunoreactive for calretinin. The reason behind this is that calretinin is transiently expressed in many cortical pyramidal neurons during development. To determine whether neurons that express or have expressed calretinin share any distinct functional characteristics, we recorded their visual response properties using GCaMP6s calcium imaging. The average orientation selectivity, size tuning, and temporal and spatial frequency tuning of this group of cells, however, match the response profile of the general neuronal population, revealing the lack of functional specialization for the features studied.

## INTRODUCTION

Calretinin (gene symbol *Calb2*) is a calcium-binding protein that took its name from its structural similarity to calbindin and to the location where it was first discovered, the retina. In the mammalian neocortex, calretinin is one of the traditionally used markers to categorize interneurons, along with other proteins such as parvalbumin and somatostatin (Gonchar and Burkhalter, 1997). Indeed, in adult rodents, most or perhaps all calretinin-immunoreactive (CR-IR) neurons in the cerebral cortex stain positive for the inhibitory neurotransmitter GABA (100%, Kubota et al., 1994; >93%, Gonchar and Burkhalter, 1997; 100%, Gonchar et al., 2008). In layer II/III of mouse primary visual cortex, CR-IR neurons are even the most abundant class of GABAergic neurons (41%, Gonchar et al., 2008). The exclusively GABAergic nature of cortical CR-IR neurons is not conserved across mammals, though, as in monkey prefrontal and human temporal neocortex about a quarter of the CR-IR neurons are not positive for GABA (del Río and DeFelipe, 1996; Melchitzky et al., 2005). Still, in both monkey and rodent, the class of CR-IR neurons contains morphologically similar groups, in particular the double-bouquet neurons of layer II/III and a subpopulation of Cajal-Retzius neurons in layer I (Condé et al., 1994; see also Barinka and Druga, 2010 for a review).

Despite their abundance, surprisingly little is known about the function of CR-IR cells. The only indirect functional information comes from two anatomical findings. First, calretinin interneurons mainly target the dendrites of other GABAergic neurons in the visual cortex in both rat (Gonchar and Burkhalter, 1999) and monkey (Meskenaite, 1997). They can thus exert a disinhibitory effect on pyramidal neurons. For this reason, CR-IR interneurons are hypothesized to be gating cells (Callaway, 2004) and necessary for persistent activity (Wang et al., 2004). Second, layer I calretinin interneurons are targeted by feedback connections from higher order visual areas (Gonchar and Burkhalter, 2003). They are thus well-positioned to convey feedback information like attentional signals and scene interpretation (Lamme and Roelfsema, 2000). Other than that in rodent cortex CR-IR cells are not fast-spiking (Kawaguchi and Kubota, 1997; Porter et al., 1998; Kawaguchi and Kondo, 2002), we have no physiological information about their function and response properties.

For this reason, our original aim was to study the receptive field properties of CR-IR neurons in the mouse primary visual cortex using calcium imaging. To achieve this, we crossed the CR-ires-Cre mouse line, which expresses the enzyme Cre-recombinase in a fashion similar to endogenous CR-expression (Taniguchi et al., 2011), to a Cre-dependent reporter mouse line expressing the red fluorescent protein tdTomato (Madisen et al., 2010). Rather than marking the current situation of CR expression, the tdTom label will not only be present in all cells that express the *Calb2* gene, but also in all cells that have expressed *Calb2* in their past. Although, in many other interneuron Cre-mouse lines these two populations are not too distinct (Taniguchi et al., 2011), they are not necessarily the same. Using immunohistochemistry and the Allen Institute’s *in situ* hybridization data (Lein et al., 2007; Oh et al., 2014), we investigated to which extent these classes overlap, and found that there is extensive transient expression of CR in excitatory neurons during development. The group of tdTomato-positive (tdTom+) cells is thus far larger than the CR-IR interneurons that we originally set out to study. Calcium imaging in these animals, with the genetically encoded calcium indicator GCaMP6s (Chen et al., 2013) under the neuronal synapsin promoter, showed the tdTom+ neurons to be a large heterogeneous group with response properties similar to the general, tdTomato-negative (tdTom-) population.

## MATERIALS AND METHODS

### ANIMALS AND ANESTHESIA

We used male and female, 2–4 months old mice from a cross of homozygous B6; 129S6-Gt(ROSA)26Sor^tm14(CAG-tdTomato)Hze^/J mice (Madisen et al., 2010; Jackson Laboratories), in which a Cre-dependent transgene encoding the tdTomato fluorescent protein is inserted in the ROSA26 locus, with homozygous B6(Cg)-Calb2^tm1(cre)Zjh^/J (Taniguchi et al., 2011; Jackson Laboratories) mice expressing Cre-recombinase following the pattern of *Calb2* expression. For the surgeries (viral injection and window implantation), we anesthetized the mice with isoflurane (1.5–2.5% vol/vol) and administered three subcutaneous injections: dexamethasone (4 mg/kg), metacam (1 mg/kg), amoxicillin (100 mg/kg). We assessed the depth of anesthesia with the pedal reflex. To protect the mice’ eyes, we used cavasan ointment. During two-photon calcium imaging, the animals were anesthetized with 0.5–1.5% vol/vol isoflurane. We adjusted the flow rate depending on the response level of the animal in order to have the lowest percentage to keep the animal in an anesthetized state. The temperature of the mouse was maintained at 37°C with heating pad and rectal probe during both surgeries and recordings. All animals were kept in a 12 h day/night cycle with access to food and water ad libitum. Experiments were carried out during the day cycle. All experiments were approved by the institutional animal care and use committee of the Royal Netherlands Academy of Arts and Sciences.

### SURGICAL PROCEDURES

For the detection of the calcium changes, we used the genetically encoded calcium indicator GCaMP6s (Chen et al., 2013). Mice were injected in the right V1 (stereotactic coordinates: 2.9 mm lateral, 0.4 mm anterior to lambda), at a depth of 400 μm, with 80 nl of a solution containing the virus AAV1.Syn.GCaMP6s.WPRE.SV40 (virus titer 3.04 × 10^13^ gc/ml, University of Pennsylvania Vector Core) using a Nanoject volume injection pump (Drummond Scientific Company). Two weeks after the viral injection, the mice were anesthetized and surgically implanted with a glass window over a V1 craniotomy (Van Versendaal et al., 2012). At the start of the surgery, the scalp was anesthetized with Xylocaine and removed. Then a coated iron ring was attached with Loctite 454 over V1 to the bone parallel to the plane of the skull and sealed with black dental cement to reduce the amount of light from the monitor entering the microscope. A craniotomy was drilled with a 2 mm diameter and after opening the brain was kept moist with artificial cerebro-spinal fluid (ACSF), consisting of a solution of 125 NaCl, 10 Hepes, 5 KCl, 2 MgSO_4_, 2 CaCl_2_, and 10 Glucose, in mM. The space between the dura and the 5 mm glass window was filled with silicon oil (∼10 mPa ⋅ s viscosity, DC 200, Fluka Analytical, UK) and sealed with a type 1 glass coverslip 100 μm thickness fixed with dental cement. With dental cement also a well was created to contain the water for the immersion objective of the microscope. We started the imaging sessions 10 days after the surgery. We generally did not experience any tissue growth under the glass window. The window was cleaned before the imaging session with 70% ethanol.

### TWO-PHOTON CALCIUM IMAGING

For imaging we used a converted Olympus BX61WI confocal microscope equipped with a Ti-sapphire laser (Mai-Tai, Spectra-physics, CA, USA), with two non-descanned PMTs with filters optimized for GFP and RFP (Semrock BrightLine FF01-520/70, FF01-625/90, and FF555-Di03 dichroic). The mice were head-fixed under the objective using a magnetic holder connected to the metal ring previously implanted over the skull of the animal (see surgical procedures). The magnet^1^ had the following specifications: 21 mm outer diameter, 15 mm inner diameter at top, 9 mm inner diameter bottom, 2 mm thick^2^. A black cloth was used to cover the objective in order to prevent the light coming from the monitor to reach the PMTs. Two-photon laser scanning microscopy was performed using a wavelength of 910 nm and neurons were imaged using a 20× water-immersion objective (Olympus, 0.95 NA). We scanned at seven frames per second. Time series recordings of these neurons were performed while showing visual stimuli.

### VISUAL STIMULATION

Stimuli were presented on a gamma-corrected Dell UltraSharp U2312HM 23′′ full HD LCD monitor, placed 15 cm in front of the mouse and oriented toward the contralateral eye. Stimuli were made with custom-made Matlab scripts, available at , a fork from code written by Steve Van Hooser, and employed the PsychoPhysics Toolbox 3 (Kleiner et al., 2007). We first measured orientation tuning, using full screen square-wave drifting gratings with different directions going in steps of 30°. Unless otherwise mentioned, the stimulus duration was 2 s, the interstimulus was an isoluminant gray screen of 3 s, contrast was 90%, temporal frequency was 2 Hz and spatial frequency 0.05 cpd. For each test, stimuli were repeated until 5 min of imaging was reached, i.e., 4–5 repetitions for each stimulus. Circular ROIs were drawn around the cells, and one responsive tdTom+ cell was chosen for which subsequent stimuli were optimized. The analysis was constrained to neurons with similar stimulus preferences. Usually, there were a number of such cells per field of view. The center of the receptive field of a neuron was assessed by presenting a drifting grating of the preferred direction in one of 6 × 3 grid locations on the monitor. Next, a size tuning stimulus was shown centered at the center-of-mass of the responses of the chosen neuron to all patches, at its optimal direction, 20–40–60–80–100° of visual angle, 2 Hz and 0.05 cpd. Spatial frequency tuning curve was assessed using a full screen sinusoidal stimulus of 0.01, 0.021, 0.044, 0.092, 0.191, 0.4 cpd at the optimal orientation for the chosen neuron. Determining temporal frequency tuning was done with a full screen sinusoidal grating drifting at 1, 3, 8, 12, 16, 20 Hz.

### ANALYSIS OF CALCIUM SIGNALS

Circular ROIs were drawn centered in all cells expressing GCaMP6s using custom Matlab scripts. The changes in fluorescence were divided by the average fluorescence just before stimulus onset to obtain ΔF/F. Response was defined as the average ΔF/F from 0.5 s after stimulus onset to stimulus offset. Cells were said to be responsive if a one-sided *t*-test of responses versus baseline fluorescence was significant at the 0.1 level. Only cells that were responsive and had a maximum response of at least 5% were included in the analysis. Orientation selectivity index was defined as OSI = √{[ΣR(φ)sin(2φ)]^2^ + [ΣR(φ)cos(2φ)]^2^}/ΣR(φ), where φ is the angle of the stimulus and R(φ) the neuron’s response to it. This is equal to 1 – circular variance. Direction selectivity index (DSI) was defined by DSI = √{[ΣR(φ)sin(φ)]^2^ + [ΣR(φ)cos(φ)]^2^}/ΣR(φ). The suppression index was defined as the (Rp-Rl)/Rp where Rp is the response to the smallest stimulus that reached 95% of the maximum response, and Rl the response to the largest stimulus (Ayaz et al., 2013). Using the red channel, cells expressing tdTomato were identified. One tdTom+ cell was chosen to optimize the stimuli, but for calculating population responses for the subsequent stimuli, we selected only cells whose preferred orientation differed 30° or less from the presented orientation, and had a receptive field center within 100 pixels of the center of the size stimulus. For the spatial frequency tuning we used slightly different criteria in that we selected cells whose preferred orientation differed 60° or less from the presented orientation.

### IMMUNOHISTOCHEMISTRY AND *IN SITU* HYBRIDIZATION

After an overdose of pentobarbital (100 mg/kg i.p.), we transcardially perfused the mice with 4% paraformaldehyde (PFA) in phosphate buffered saline (PBS), and post-fixated the brains for 2 h in PFA at 4°C. After changing the brain to a PBS solution, we cut the brains in sagittal or coronal slices of 50 μm thickness. We incubated the slices for 2 h in 500 μl blocking solution (0.1% Triton X-100, 5% NGS in PBS) on a rotary shaker at room temperature. We then incubated the slices in 250 μl of primary antibody per well and left it overnight at 4°. The next day we discarded the primary antibody solution and proceeded with three washes of 10 min at room temperature on the rotary shaker with 500 μl of washing solution (0.1% Tween in PBS). We added 250 μl per well of the secondary antibody solution and incubated for 1 h at room temperature on the rotary shaker. We washed the slices in washing solution three times for 10 min at room temperature on the rotary shaker.

We used the following primary antibodies: (1) mouse anti-calretinin, Millipore (1:700 in blocking solution); (2) rabbit anti-parvalbumin, Swant (1:1000 in blocking solution); (3) rat anti-somatostatin, Millipore (1:200 in blocking solution); (4) mouse anti-SatB2, Santa Cruz (1:1000 in blocking solution). As secondary antibodies we used: (1) Goat anti-mouse Alexa 647, Invitrogen (1:700); (2) Goat anti-rabbit Alexa 488, Life technologies (1:700); (3) Goat anti-rat Alexa 647, Invitrogen (1:700); (4) goat anti-mouse Alexa 488, Invitrogen (1:700). Stained sections were mounted on glass slides with mowiol.

For the imaging of the immunostained sections we used a Leica TCS SP5 Confocal microscope and we mainly imaged the superficial layers of primary visual cortex. *In situ* hybridization image for *Calb2* expression was retrieved from the Allen Mouse Brain Atlas, available from^3^ (Lein et al., 2007). The *in situ* hybridization images for the CR-ires-Cre and Ai14 cross are collected from the Allen Mouse Brain Connectivity Atlas, available from^4^ (Oh et al., 2014).

### STATISTICS

Values in the text are expressed as the mean ± SEM. For the population statistics of comparing the response properties of tdTom+ and tdTom- cells, the distribution were first tested for normality with the Shapiro–Wilk test. In all cases, at least one of the distribution failed and we performed the non-parametric Mann–Whitney *U* test (Kruskal–Wallis).

## RESULTS

### OVERLAP OF tdTOMATO AND CALRETININ EXPRESSION

To study the function in visual processing of neurons that express calretinin, we used offspring from a cross of two mouse lines: one expressing the tdTomato red fluorescent protein inside the *Rosa26* locus preceded by a floxed stop cassette; and the CR-ires-Cre line in which the *Cre-recombinase* coding sequence follows the *Calb2* promoter and an IRES-element. This resulted in the expression of the tdTom protein in all the cells that sufficiently expressed *Calb2* and thus Cre during their existence.

**FIGURE 1:**
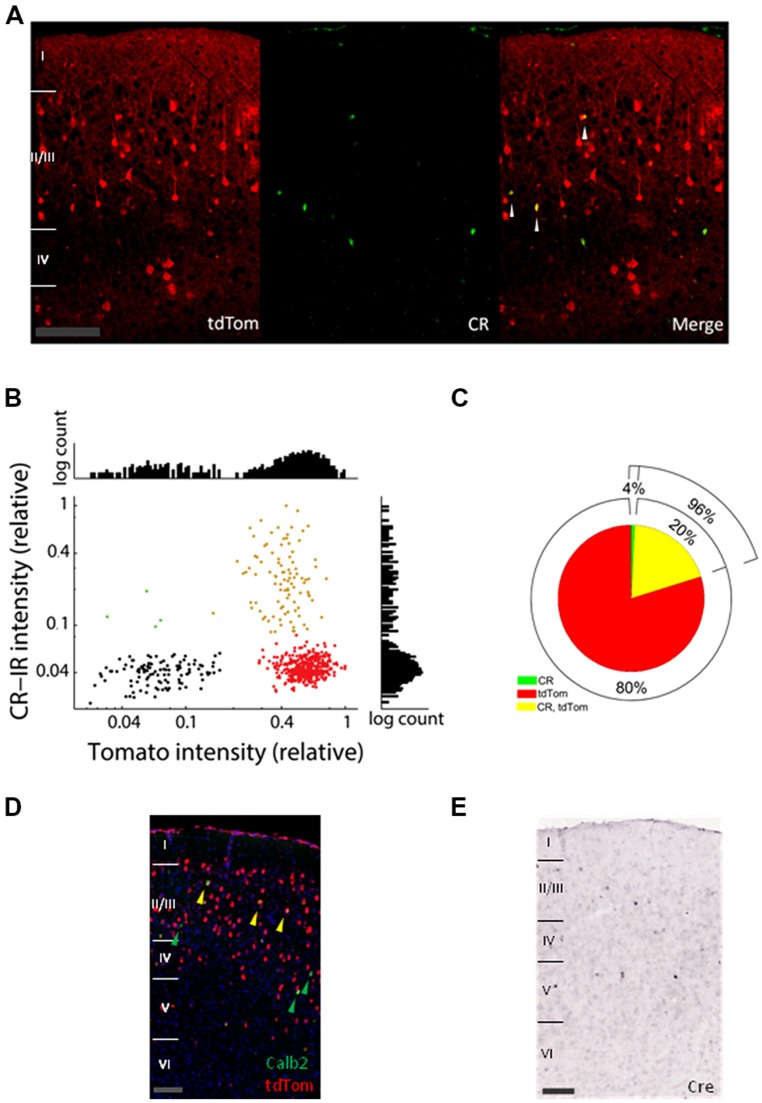
**Expression of tdTomato does not match mRNA and protein expression of calretinin. (A)** Confocal images of coronal slices of V1 from a tdTomato× CR-ires-Cre mouse immunostained with Millipore mouse Calretinin (CR) antibody (green). White arrows show colocalization of CR and tdTom. Scale bar is 100 μm. **(B)** Average label intensities for CR-IR and tdTom+ expressing cells. Red points are from cells classified as tdTom+, green points belong to CR-IR cells, and yellow belong to cells where both proteins colocalize. Black shows a collection of background expression levels. Intensity is normalized per slice to the maximum average intensity found for any cell in each slice. Graphs at right and top show histograms of the intensities for the *y*- and *x*-axis, respectively. **(C)** Pie graph showing the colabeling of CR immunoreactive and tdTomato protein. **(D)** *In situ* hybridization images of V1 from a P56 mouse from the same cross (Allen Mouse Brain Connectivity Atlas, experiment 267223174, image 31). In red tdTomato mRNA expressing cells, in green cells expressing the *Calb2* mRNA (green arrows) and in yellow colocalization of the two mRNAs (yellow arrows). Scale bar is 100 μm. **(E)** *In situ* hybridization of Cre-recombinase mRNA in P56 visual cortex is consistent with CR expression (image from Allen Mouse Brain Atlas, experiment 100146208, image 31). Scale bar is 100 μm.

First, we investigated to which extent the cells expressing tdTomato (tdTom+ cells) overlap with the Calretinin immunoreactive cells (CR-IR cells). In **Figure 1A**, tdTom+ cells are shown in red and the CR-IR cells in green in a slice of adult V1. The levels of CR immunoreactivity varied among the positive cells, but showed a bimodal distribution separated at about 8% of the most intense levels (**Figure 1B**). Although it was possible that even for the very low levels of labeling, there was some calretinin expression, it did suggest a natural classification of CR-IR positive and negative cells. The distribution of tdTomato expression levels was even more clearly bimodal and made a clear distinction between tdTom+ and tdTom- negative cells possible (**Figure 1B**). Using these classifications, we estimated that only 20% of tdTom+ cells were positive for calretinin (**Figure 1C**). By contrast, 96% of the CR-IR cells expressed tdTomato. To understand whether the protein expression differences between CR and tdTomato came from a difference of translation of the proteins, we checked the Allen Brain Atlas, where *in situ* hybridization images are available for the CR-ires-Cre mice (Oh et al., 2014). A co-hybridization for tdTomato mRNA and CR mRNA showed a pattern similar to the protein immunohistochemistry images (**Figure 1D**). This suggested that the differences were not due to translational regulation of CR or tdTomato. Indeed, *in situ* hybridization of Cre-recombinase mRNA showed a pattern remarkably similar to the expression pattern of *Calb2* mRNA and CR protein (**Figure 1E**). Cre-recombinase is thus not expressed at P56 in most of the tdTom+ cells. It must have been expressed earlier in the history of these cells, when it irreversibly activated the *tdTomato* gene. Together with the reported high specificity of the CR-ires-Cre line when checked as adults with a Cre-dependent marker virus (Taniguchi et al., 2011), this shows that the tdTom+ cells that are not CR-immunoreactive in the adult mouse have transiently expressed CR in their past.

### tdTOMATO COLOCALIZES WITH DIFFERENT INTERNEURONAL AND PYRAMIDAL MARKERS

**FIGURE 2:**
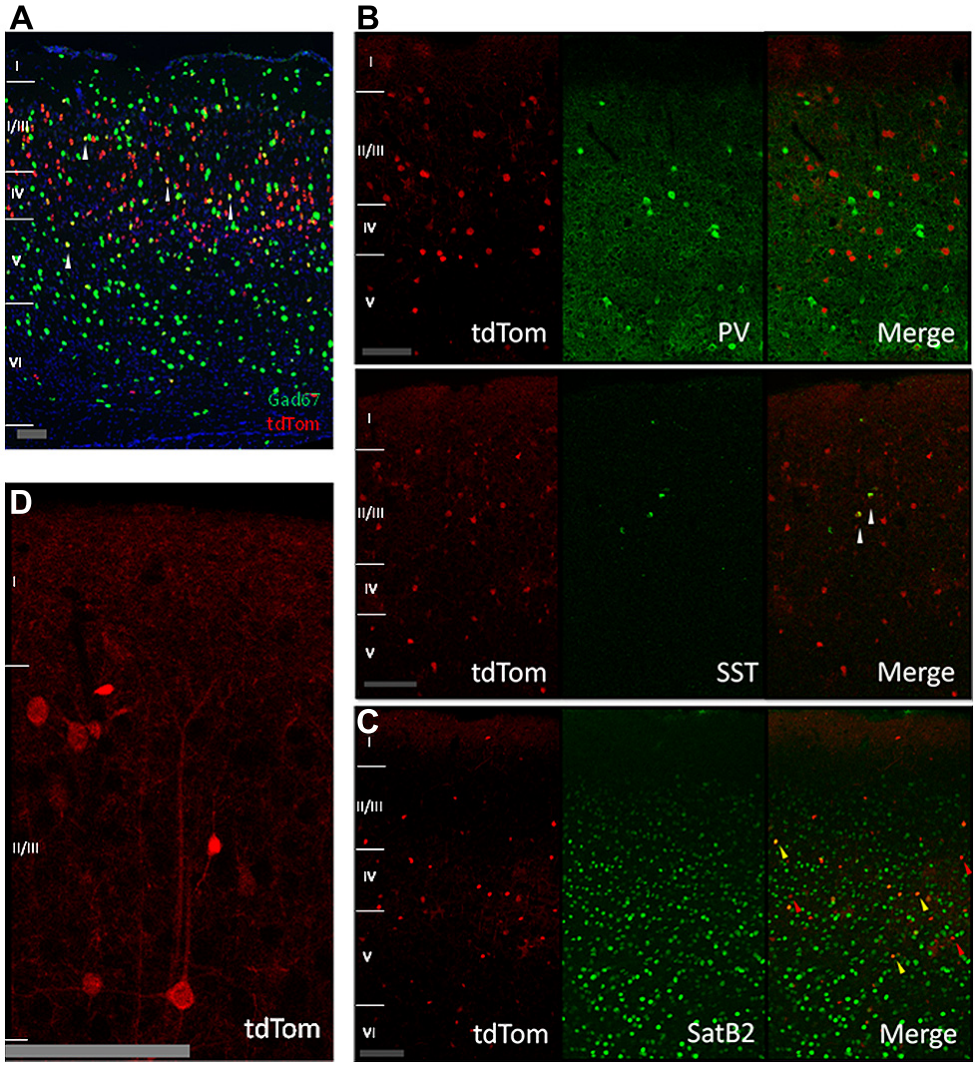
**Cre-recombinase in inhibitory and pyramidal neurons. (A)** *In situ* hybridization image showing tdTomato expressing neurons in red, *Gad67* positive neurons in green, and in yellow neurons expressing both mRNAs (highlighted by white arrows; image from Allen Mouse Brain Connectivity Atlas, experiment 180304494, image 33). Scale bar is 100 μm. **(B)** Expression of tdTomato in other inhibitory neurons. First row: immunostained sagittal sections from V1 showing no colocalization between tdTom and parvalbumin (PV). Second row: immunostained sagittal sections from V1 showing low colocalization of tdTom and somatostatin (SST) protein (white arrow). Scale bar is 100 μm. **(C)** Expression of tdTomato in pyramidal neurons. Immunostained coronal slices from V1 for the pyramidal marker SatB2. Red arrows show cells that are positive only for tdTomato, yellow arrows show cells that express both proteins. Scale bar is 100 μm. **(D)** Close up of a tdTomato neuron with a pyramidal shape. Scale bar is 100 μm.

We next wanted to understand the nature of the tdTomato-positive cells, which clearly contained more cells than just the CR-IR interneurons. The *in situ* hybridization data of the Allen Institute for this mouse cross showed a low colocalization between the tdTomato and the GABA synthesizing enzyme *Gad67* mRNAs (**Figure 2A**, *Gad67* shown in green). We wondered if the overlap of tdTomato with *Gad67* was completely due to CR expressing interneurons or if parvalbumin-positive or somatostatin-positive interneuron classes had a transient expression of CR during their development or migration. Our immunostainings showed no colocalization with the parvalbumin protein and a low colocalization with somatostatin (**Figure 2B**). This showed that parvalbumin interneurons do not transiently express CR. The fraction of somatostatin neurons that expressed tdTomato was consistent with previous reports of SST interneurons with CR immunoreactivity (Xu et al., 2006; Gonchar et al., 2008). Given the relatively low overlap with *Gad67*, we wondered whether the tdTomato positive cells were pyramidal neurons and stained for SatB2 which labels a large group of pyramidal cells (Britanova et al., 2008). We found that indeed 60% of tdTom+ cells are SatB2 positive and 5% of SatB2-IR cells are positive for tdTomato (**Figure 2C**, quantification not shown). Thus, we conclude that most of the tdTom cells are pyramidal neurons, and indeed many tdTom+ cells have pyramidal morphology (for example, **Figure 2D**).

### VISUAL RESPONSE PROPERTIES OF tdTOM+ NEURONS MATCH THOSE OF tdTOM- NEURONS

Although the tdTom+ cells formed a larger group than the CR interneurons, the cells all expressed calretinin in their past, and this may be an indication of not only a common history but also of a common functional role. To explore whether they showed any specific response to visual stimulation, we imaged the visual response properties of tdTom+ cells in primary visual cortex using two-photon imaging of the genetically encoded calcium indicator GCaMP6s (Chen et al., 2013), virally expressed behind the neuronal *Synapsin* promoter.

**FIGURE 3:**
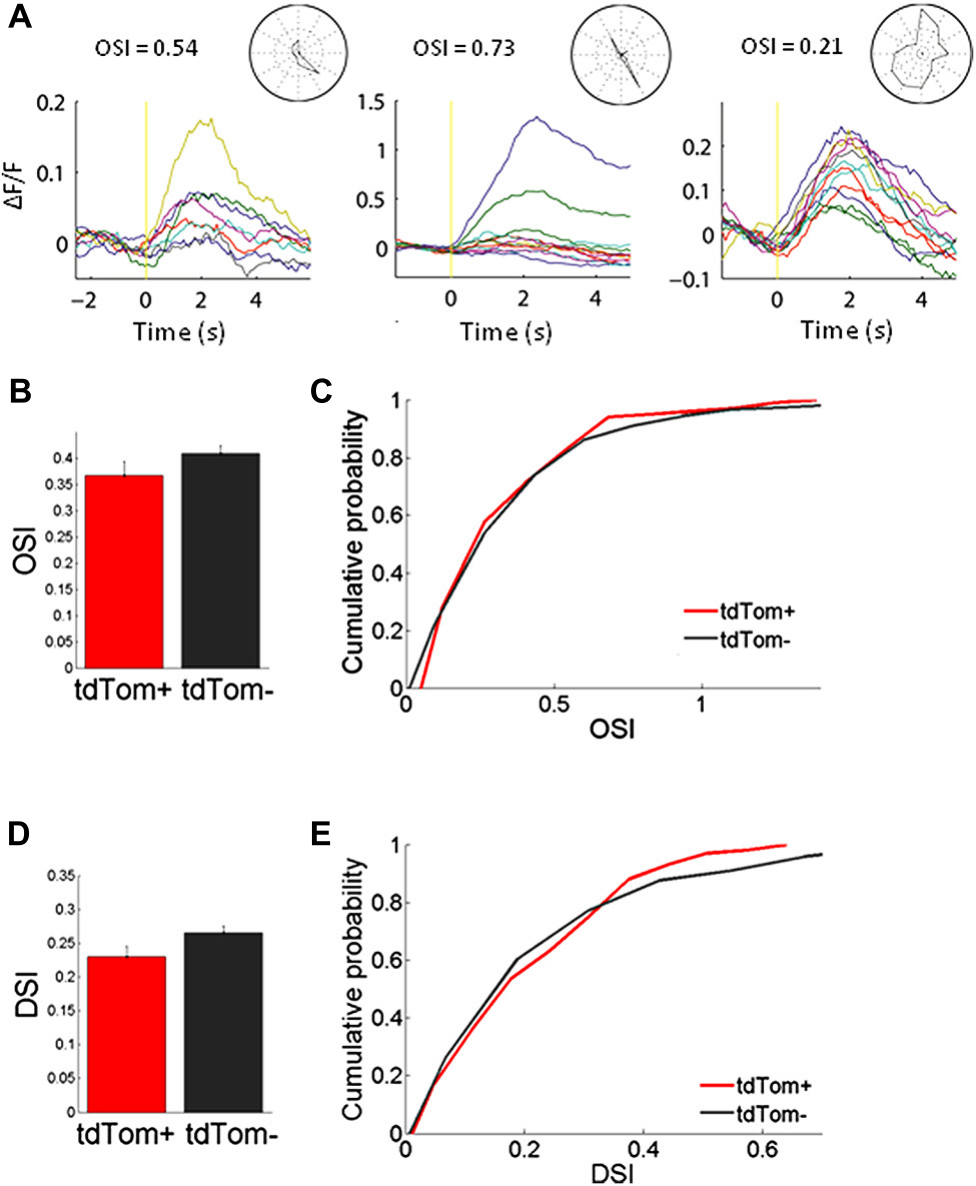
**Orientation tuning of tdTom+ neurons is like the general population. (A)** Example peristimulus time histograms for tdTom+ neurons during visual stimulation with drifting gratings. Yellow vertical line represents the start of the visual stimulation. Normalized fluorescence changes to different grating orientations are shown by different colors. Stimulus duration is 2 s. **(B)** No difference in mean orientation selectivity index (OSI) for the tdTom+ and tdTom- neurons. Error bars show SEM. **(C)** Distributions of OSI are equal for tdTom+ and tdTom- neurons. **(D)** No difference in mean direction selectivity index (DSI). Error bars show SEM. **(E)** Direction selectivity index distributions of tdTom+ and tdTom- population are not different.

**FIGURE 4:**
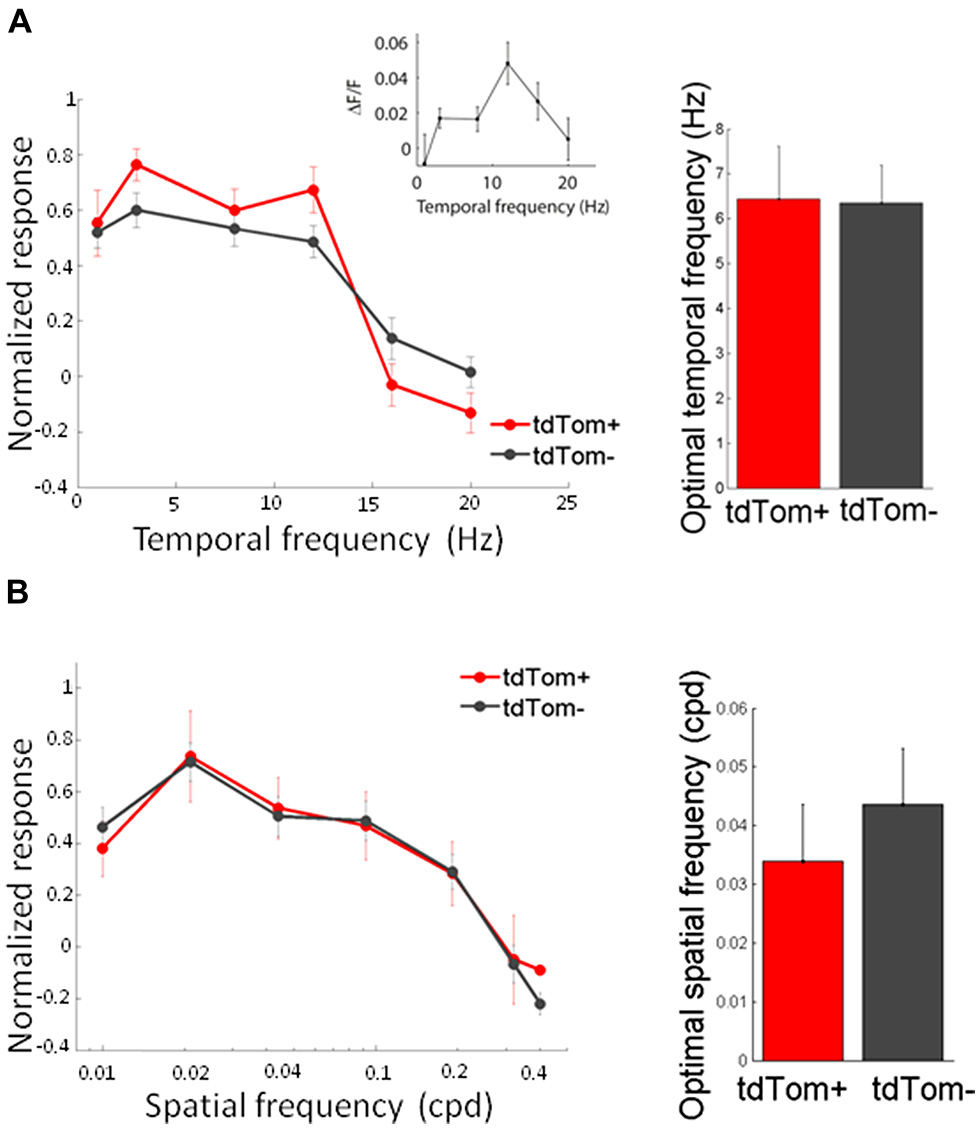
**Temporal and spatial frequency tuning of tdTom+ neurons is like the general population. (A)** Left, population average tuning to temporal frequency are not different for tdTom+ and tdTom- groups. Inset, example tuning of a tdTom+ neuron. Right, mean optimal temporal frequencies are identical. Error bars show SEM. **(B)** Left, population response tuning to spatial frequency is the same for tdTom+ and tdTom- groups. Right, mean optimal spatial frequencies are not different. Error bars show SEM.

We started by measuring orientation tuning. The tdTom+ group contained a variety of orientation or direction selective and unselective cells (some examples shown in **Figure 3A**). The tuning of tdTom+ group did not stand out of the population as a whole. The tdTom+ and the tdTom- neurons were equally orientation tuned, as assessed by the orientation selectivity index, which was equal to 1-circular variance, (mean OSI = 0.37 ± 0.03, *N* = 118 and 0.41 ± 0.02, *N* = 396, respectively; *p* = 0.27, Kruskal–Wallis test, *K*[1] = 1.2; six mice, Figures 3B,C). The distribution of direction selectivity of the two groups were also very similar (mean DSI was 0.23 ± 0.02, *N* = 118 and 0.27 ± 0.01, *N* = 396 respectively; *p* = 0.31, Kruskal–Wallis test, *K*[1] = 1.0; Figures 3D,E). After showing all orientations, we picked one and varied the temporal frequency. Next, we selected the responses of all cells that had an orientation preference within 30° of the presented orientation. We found both lowpass and bandpass cells, for both the tdTom+ and tdTom- population. We found no differences in temporal frequency tuning. Both groups responded on average up to about 15 Hz (**Figure 4A**). The optimal temporal frequency was equal for the two groups (tdTom+, 6.4 ± 1.2 Hz, *N* = 16, tdTom-, 6.4 ± 0.9 Hz, *N* = 36; *p* = 0.95, Kruskal–Wallis test, *K*[1] = 0.003; four mice).

**FIGURE 5:**
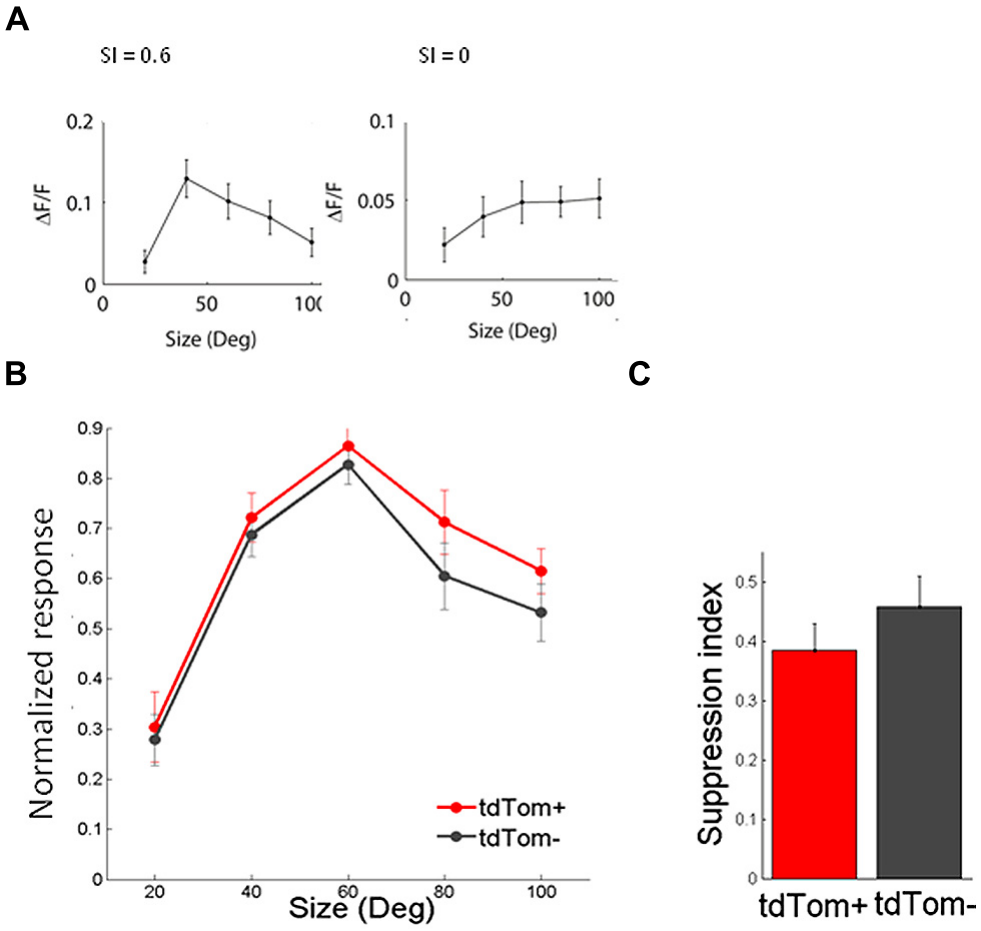
**Size tuning of tdTom+ is like the general population. (A)** Example tuning of two tdTom+ neurons to visual stimulation of a circular patch of different sizes, and their corresponding Suppression Index values. **(B)** Population average of normalized response to differently sized gratings are not different for tdTom+ and tdTom- groups. Error bars are SEM. **(C)** Mean suppression index is not different for the two groups. Error bars are SEM.

In the same way, we measured the response to sinusoidal gratings of different spatial frequencies. Also in this respect did the two populations not differ from each other. The average spatial frequency tuning curve overlapped and the mean optimal spatial frequencies were equal for tdTom+ and tdTom- (0.034 ± 0.01 cpd, *N* = 6 and 0.044 ± 0.01 cpd, *N* = 20, respectively; *p* = 0.9, Kruskal–Wallis test, *K*[1] = 0.03; in four mice, **Figure 4B**). The curves for both temporal and spatial frequency reached values slightly lower than zero at high frequencies. This was due to the slow kinetics of the GCaMP6s indicator. The 3 s of interstimulus interval following the 2 s of visual stimulation were not enough for the fluorescence to completely return to the baseline level, meaning that when the subsequent stimulus evokes no or very little response the response may appear slightly negative (see also **Figure 3A**). Because of the feedback connections from higher areas onto CR interneurons (Gonchar and Burkhalter, 2003) and the hypothesized role of these feedback connections in surround suppression (Bair et al., 2003), we were interested in the size tuning properties of the tdTom+ cells. One might expect perhaps that neurons receiving feedback from cells with larger receptive fields, or interneurons that are involved in surround suppression, show larger responses with increasing stimulus size (Adesnik et al., 2012). In the tdTom+ group, we found cells showing a strong suppression and cells that lacked suppression (**Figure 5A**). The average size tuning curve and the distribution of suppression indices did not show a difference between the tdTom+ and tdTom- neurons (mean SI 0.38 ± 0.05, *N* = 21 and 0.43 ± 0.07, *N* = 38, respectively; *p* = 0.4, *t*-test, *t*[33] = -0.90; four mice, Figures 5B,C). We conclude that in none of the studied visual response properties the tdTom+ population is significantly different from the tdTom- population.

## DISCUSSION

We studied the visual response properties of a class of labeled neurons in the primary visual cortex that resulted from a cross of a Cre-dependent tdTomato reporter line and a line in which Cre-recombinase was expressed like calretinin. These neurons that we called tdTom+ do not seem to have different response properties compared to the general population (tdTom-).

Originally, the aim of the cross was to study the function of CR-immunoreactive interneurons. However, in visual cortex, only a fifth of the tdTom+ cells showed CR-immunoreactivity, and mRNA levels showed a similarly low overlap between tdTomato and *Calb2*. In contrast, when cells were labeled in the mature CR-ires-Cre animal with a virus with a Cre-dependent fluorescent reporter vector, the specificity was as high as 91% (Taniguchi et al., 2011). *In situ* hybridization also showed that the pattern of expression of Cre-recombinase is very similar to the pattern of CR expression in these animals. We must conclude that the high number of tdTom+ cells that do not express CR is due to transient expression of CR. The tdTom+ cells have expressed CR and thus cre-recombinase in their past. This has activated the *tdTomato* transgene and the cells have expressed tdTomato ever since, even when they no longer expressed CR. There is the possibility that some or all tdTom+ cells that we judged negative for CR-IR, in fact do express calretinin at a very low level, but this is not detectable above the background level in our staining.

We investigated whether the tdTom+ cells perhaps included another set of interneurons besides the CR-interneurons. We found, however, no colocalization with parvalbumin. We did find some colocalization with somatostatin (SST), which was consistent with the reported overlap of SST and CR expressing interneuron populations (Xu et al., 2006; Gonchar et al., 2008). Available *in situ* hybridization data showed that many tdTom+ cells did not express the GABA-synthesizing enzyme *Gad67* and would thus not be interneurons. Indeed, 60% of tdTom+ neurons were positive for the pyramidal marker SatB2 and pyramidal shaped tdTom+ neurons were recognizable in confocal images.

Our findings are consistent with the report that CR immunoreactivity starts to be widely present in the cortical anlage from embryonic day 14 in rats, and continues in the first two postnatal weeks. Many of the CR-IR neurons at these early stages of development show undifferentiated non-pyramidal shapes, but there is also transient expression in some pyramidal-like neurons in layers V, VI and layer II/III (Fonseca et al., 1995). We showed that the extent of this transient expression is very high. Transient expression of CR protein in pyramidal-like neurons has also been reported in rat hippocampus during development (Jiang and Swann, 1997) and adult neurogenesis (Brandt et al., 2003).

There is a considerable sequence homology between the promoters of the mouse *Calb2* and the human *CALB2* genes (Strauss et al., 1997), and thus the regulation of CR expression in primate and mouse may be quite similar. It is thus not inconceivable that the CR-IR pyramidal neurons that have been reported in monkeys and human (del Río and DeFelipe, 1996; Melchitzky et al., 2005) are homologous to cells that are contained in our tdTom+ group.

The investigation of the visual response properties of the heterogeneous group of neurons with a shared CR-IR history revealed the absence of specificity in their response to the studied features. Unlike parvalbumin neurons, which show a reduced orientation selectivity (Kerlin et al., 2010; Hofer et al., 2011), tdTom+ cells showed an orientation selectivity identical to the general population. The mean OSI was slightly lower than the mean OSI found for excitatory neurons alone in another study. Although measurements of OSI are dependent on recording conditions, this would suggest that the group of interneurons included in our tdTom+ sample have a lower orientation selectivity than the pyramidal cell population. The tdTom+ cells also showed no more or less direction selectivity than the general population. We cannot rule out that we would have missed a small difference, but our study had about an 80% probability to detect a difference in orientation or direction selectivity of 20% with 95% confidence. A halving of the mean OSI for the tdTom+ group compared to that of the tdTom- group, like the difference that was reported for all GABAergic inhibitory neurons compared to excitatory neurons (Kerlin et al., 2010), we would have been able to detect with 100% certainty.

Previously, it was found that the spatial frequency tuning can vary with cortical cell type (Kerlin et al., 2010) and in particular putative fast-spiking interneurons show a preferred frequency only about half of that of excitatory neurons (Niell and Stryker, 2008). The tdTom+ and tdTom- groups, however, did not differ significantly in spatial frequency. We had, however, less statistical power for the spatial frequency tuning than for orientation and direction selectivity, and had only 30% chance to pick up a similar difference for the preferred spatial frequency of the two groups at the 95% confidence level. We also did not find any difference in temporal frequency tuning. We know no reference study where a difference in temporal frequency between neuron types within one area was reported, but we had a power of 80% to find a 65% difference in temporal frequency. The size tuning of tdTom+ was also not different from the other cells, unlike somatostatin neurons which show considerably less surround suppression (Adesnik et al., 2012). Our study would have had enough power to detect if there was a similarly large difference between the tdTom+ and tdTom- population, and even a 91% certainty of detecting only a halving of the suppression index as was seen for PV interneurons (Adesnik et al., 2012).

We could thus not discern any particular feature of this group of cells, other than their common CR history. The underlying reason for the common CR expression during their development remains unclear. For investigating the visual response properties of CR-IR interneurons, viral transfection with a vector for cre-dependent expression of GCaMP6s or another fluorescent protein after the second postnatal week will be needed.

## Conflict of Interest Statement

The authors declare that the research was conducted in the absence of any commercial or financial relationships that could be construed as a potential conflict of interest.

## Footnotes

1 http://www.supermagnetman.net

2 https://sites.google.com/site/alexanderheimel/protocols/magnetic-head-holder for more details.

3 http://mouse.brain-map.org

4 http://connectivity.brain-map.org

## References

[B1] Adesnik H. Bruns W. Taniguchi H. Huang Z. J. Scanziani M. (2012). A neural circuit for spatial summation in visual cortex. *Nature* 490 226–231 10.1038/nature11526 23060193 PMC3621107

[B2] Ayaz A. Saleem A. B. Schölvinck M. L. Carandini M. (2013). Locomotion controls spatial integration in mouse visual cortex. *Curr. Biol.* 23 890–894 10.1016/j.cub.2013.04.012 23664971 PMC3661981

[B3] Bair W. Cavanaugh J. R. Movshon J. A. (2003). Time course and time-distance relationships for surround suppression in macaque V1 neurons. *J. Neurosci.* 23 7690–7701 12930809 10.1523/JNEUROSCI.23-20-07690.2003 PMC6740744

[B4] Barinka F. Druga R. (2010). Calretinin expression in the mammalian neocortex: a review. *Physiol. Res.* 59 665–677 20406030 10.33549/physiolres.931930

[B5] Brandt M. D. Jessberger S. Steiner B. Kronenberg G. Reuter K. Bick-Sander A. (2003). Transient calretinin expression defines early postmitotic step of neuronal differentiation in adult hippocampal neurogenesis of mice. *Mol. Cell. Neurosci.* 24 603–613 10.1016/S1044-7431(03)00207-0 14664811

[B6] Britanova O. de Juan Romero C. Cheung A. Kwan K. Y. Schwark M. Gyorgy A. (2008). Satb2 is a postmitotic determinant for upper-layer neuron specification in the neocortex. *Neuron* 57 378–92 10.1016/j.neuron.2007.12.028 18255031

[B7] Callaway E. M. (2004). Feedforward, feedback and inhibitory connections in primate visual cortex. *Neural Netw.* 17 625–632 10.1016/j.neunet.2004.04.004 15288888

[B8] Chen T. W. Wardill T. J. Sun Y. Pulver S. R. Renninger S. L. Baohan A. (2013). Ultrasensitive fluorescent proteins for imaging neuronal activity. *Nature* 499 295–300 10.1038/nature12354 23868258 PMC3777791

[B9] Condé F. Lund J. S. Jacobowitz D. M. Baimbridge K. G. Lewis D. A. (1994). Local circuit neurons immunoreactive for calretinin, calbindin D-28k or parvalbumin in monkey prefrontal cortex: distribution and morphology. *J. Comp. Neurol.* 341 95–116 10.1002/cne.903410109 8006226

[B10] del Río M. R. DeFelipe J. (1996). Colocalization of calbindin D-28k, calretinin, and GABA immunoreactivities in neurons of the human temporal cortex. *J. Comp. Neurol.* 369 472–482 10.1002/(SICI)1096-9861(19960603)369:3<472::AID-CNE11>3.0.CO;2-K 8743426

[B11] Fonseca M. Dél Rio J. A. Martinez A. Gómez S. Soriano E. (1995). Development of calretinin immunoreactivity in the neocortex of the rat. *J. Comp. Neurol.* 361 177–192 10.1002/cne.903610114 8550878

[B12] Gonchar Y. Burkhalter A. (1997). Three distinct families of GABAergic neurons in rat visual cortex. *Cereb. Cortex* 7 347–358 10.1093/cercor/7.4.347 9177765

[B13] Gonchar Y. Burkhalter A. (1999). Connectivity of GABAergic calretinin-immunoreactive neurons in rat primary visual cortex. *Cereb. Cortex* 9 683–696 10.1093/cercor/9.7.683 10554991

[B14] Gonchar Y. Burkhalter A. (2003). Distinct GABAergic targets of feedforward and feedback connections between lower and higher areas of rat visual cortex. *J. Neurosci.* 23 10904–10912 14645486 10.1523/JNEUROSCI.23-34-10904.2003 PMC6740993

[B15] Gonchar Y. Wang Q. Burkhalter A. (2008). Multiple distinct subtypes of GABAergic neurons in mouse visual cortex identified by triple immunostaining. *Front. Neuroanat.* 1:3 10.3389/neuro.05.003.2007 PMC2525923 18958197

[B16] Hofer S. B. Ko H. Pichler B. Vogelstein J. Ros H. Zeng H. (2011). Differential connectivity and response dynamics of excitatory and inhibitory neurons in visual cortex. *Nat. Neurosci.* 14 1045–1052 10.1038/nn.2876 21765421 PMC6370002

[B17] Jiang M. Swann J. W. (1997). Expression of calretinin in diverse neuronal populations during development of rat hippocampus. *Neuroscience* 81 1137–1154 10.1016/S0306-4522(97)00231-5 9330374

[B18] Kawaguchi Y. Kondo S. (2002). Parvalbumin, somatostatin and cholecystokinin as chemical markers for specific GABAergic interneuron types in the rat frontal cortex. *J. Neurocytol.* 31 277–287 10.1023/A:1024126110356 12815247

[B19] Kawaguchi Y. Kubota Y. (1997). GABAergic cell subtypes and their synaptic connections in rat frontal cortex. *Cereb. Cortex* 7 476–486 10.1093/cercor/7.6.476 9276173

[B20] Kerlin A. M. Andermann M. L. Berezovskii V. K. Reid R. C. (2010). Broadly tuned response properties of diverse inhibitory neuron subtypes in mouse visual cortex. *Neuron* 67 858–871 10.1016/j.neuron.2010.08.002 20826316 PMC3327881

[B21] Kleiner M. Brainard D. Pelli D. (2007). What’s new in Psychtoolbox-3? *Perception* 36:14 10.1068/v070821

[B22] Kubota Y. Hattori R. Yui Y. (1994). Three distinct subpopulations of GABAergic neurons in rat frontal agranular cortex. *Brain Res.* 649 159–173 10.1016/0006-8993(94)91060-X 7525007

[B23] Lamme V. A. Roelfsema P. R. (2000). The distinct modes of vision offered by feedforward and recurrent processing. *Trends Neurosci.* 23 571–579 10.1016/S0166-2236(00)01657-X 11074267

[B24] Lein E. S. Hawrylycz M. J. Ao N. Ayres M. Bensinger A. Bernard A. (2007). Genome-wide atlas of gene expression in the adult mouse brain. *Nature* 445 168–176 10.1038/nature05453 17151600

[B25] Madisen L. Zwingman T. A. Sunkin S. M. Oh S. W. Zariwala H. A. Gu H. (2010). A robust and high-throughput Cre reporting and characterization system for the whole mouse brain. *Nat. Neurosci.* 13 133–140 10.1038/nn.2467 20023653 PMC2840225

[B26] Melchitzky D. S. Eggan S. M. Lewis D. A. (2005). Synaptic targets of calretinin-containing axon terminals in macaque monkey prefrontal cortex. *Neurosci.* 130 185–195 10.1016/j.neuroscience.2004.08.046 15561434

[B27] Meskenaite V. (1997). Calretinin-immunoreactive local circuit neurons in area 17 of the cynomolgus monkey, Macaca fascicularis. *J. Comp. Neurol.* 379 113–132 10.1002/(SICI)1096-9861(19970303)379:1<113::AID-CNE8>3.0.CO;2-7 9057116

[B28] Niell C. M. Stryker M. P. (2008). Highly selective receptive fields in mouse visual cortex. *J. Neurosci.* 28 7520–7536 10.1523/JNEUROSCI.0623-08.2008 18650330 PMC3040721

[B29] Oh S. W. Harris J. A. Ng L. Winslow B. Cain N. Mihalas S. (2014). A mesoscale connectome of the mouse brain. *Nature* 508 207–214 10.1038/nature13186 24695228 PMC5102064

[B30] Porter J. T. Cauli B. Staiger J. F. Lambolez B. Rossier J. Audinat E. (1998). Properties of bipolar VIPergic interneurons and their excitation by pyramidal neurons in the rat neocortex. *Eur. J. Neurosci.* 10 3617–3628 10.1046/j.1460-9568.1998.00367.x 9875341

[B31] Strauss K. I. Kuźnicki J. Winsky L. Kawagoe J. I. Hammer M. Jacobowitz D. M. (1997). The mouse calretinin gene promoter region: structural and functional components. *Brain Res. Mol. Brain Res.* 49 175–187 10.1016/S0169-328X(97)00143-5 9387877

[B32] Taniguchi H. He M. Wu P. Kim S. Paik R. Sugino K. (2011). A resource of cre driver lines for genetic targeting of GABAergic neurons in cerebral cortex. *Neuron* 71 995–1013 10.1016/j.neuron.2011.07.026 21943598 PMC3779648

[B33] Van Versendaal D. Rajendran R. Saiepour M. H. Klooster J. Smit-Rigter L. Sommeijer J. P. (2012). Elimination of inhibitory synapses is a major component of adult ocular dominance plasticity. *Neuron* 74 374–83 10.1016/j.neuron.2012.03.015 22542189

[B34] Wang X. J. Tegnér J. Constantinidis C. Goldman-Rakic P. S. (2004). Division of labor among distinct subtypes of inhibitory neurons in a cortical microcircuit of working memory. *Proc. Natl. Acad. Sci. U.S.A.* 101 1368–1373 10.1073/pnas.0305337101 14742867 PMC337059

[B35] Xu X. Roby K. D. Callaway E. M. (2006). Mouse cortical inhibitory neuron type that coexpresses somatostatin and calretinin. *J. Comp. Neurol*. 499 144–160 10.1002/cne.21101 16958092

